# Abnormalities of the Blood.—The Etiology of Asthma

**Published:** 1899-02

**Authors:** George N. Jack

**Affiliations:** Depew, N. Y.


### BUFFALO MEDICAL JOURNAL.

VOL. XXXVIII. FEBRUARY, 1899. No. 7.

### Original Communications.

### ABNORMALITIES OF THE BLOOD.—THE ETIOLOGY OF ASTHMA.

BY GEORGE N. JACK, M. D., Depew, N. Y.

ASTHMA is generally considered by the profession as an uninteresting and hopeless subject. A disease that comes as a sort of a *sine qua non*; to remain with its poor victim through life, and when death mercifully closes the strife, to mysteriously disappear, with all its instruments of torture, thus leaving no marks behind by which the pathologist may discover any clue as to the cause.

When I was an undergraduate, I heard a physician of some prominence tell a mother, who had brought to him her child suffering with asthma, that he did not pretend to know much about the disease, and that he did not think there was any help for it. While in college I heard one of my teachers state that he believed asthma was a curable disease, but he admitted that he did not see many cases, as a certain advertising physician got hold of most of them and wrought a permanent cure somehow, he did not know how, but he thought it was accomplished largely by the use of arsenic, potassium iodide and attention to the reflexes.

I believe it is time that the profession had some positive knowledge of a disease so important as asthma, that it may be scientifically and successfully treated, and with this aim in view this paper was written.

Let us consider asthma as a disease caused by some abnormal condition of the blood which reduces its oxygen absorbing and carrying property, thus causing a strained respiratory effort. The respiratory center of the medulla being irritated by this poorly oxygenated

1. Read before the Buffalo Academy of Medicine, December 13, 1898.

blood, telegraphs down the pneumogastric for more oxygen. It being the function of the lungs to supply this oxygen, they are vigorously set to work, forcibly expanding and contracting, until the bronchioles and the air sacs are filled to their utmost with air. Yet the blood not being able to absorb or carry this oxygen, still reaches the respiratory center in the same impoverished condition, which center responds as before and stimulates the respiratory muscles to a still greater strained effort. The excessive amount of air in the meantime and the strained condition of the bronchioles and air sacs, has resulted in the production of an acute hyperemia of their mucous membranes, which further aggravates and increases the severity of the attack. This state of affairs continues with all the characteristic symptoms of asthma, until the blood undergoes some chemical or physical change, which permits of a more rapid and normal absorption of oxygen. Each attack, according to its severity and duration, leaves some permanent or temporary pathological lesion, either upon the lungs, heart, alimentary tract, *i.e.*, fermentation or decomposition of food from an insufficient supply of oxygen, or an increased impoverishment of the blood, which condition or conditions predispose to other attacks, and thus the asthmatic state is kept up until by change of diet, surroundings and location, or by the proper administration of medicines, the blood is once again restored to its normal condition.

I am so firm a believer that asthma is produced by some dyscrasia of the blood differing in different cases, even in the same patient, as to age and surroundings, to truthfully believe that no physician should be allowed to prescribe for a case without first procuring a careful analysis of the blood. This is not a hard statement to prove, when one considers the relationship and co-workmanship of the lungs and the blood. It being the function of the lungs to carry oxygen to the blood, and of the blood to take oxygen from the lungs, then having impaired oxygenation with no pathological condition of the lungs as has always been proved in asthmatics, where can the pathological state exist if not with the blood?

That the lungs should spasmodically contract and expel a substance that cannot be utilised is only a natural phenomenon. This phenomenon is most beautifully illustrated by the stomach, for, as we all know, the stomach may be in a perfectly normal condition and contain food that it usually most rapidly assimilates, yet if the digestive ferments are absent or the liver or intestines in an unfit state to receive its contents or any more nourishment, it does not take the stomach long to forcibly contract and expel its contents. The similarity between the lungs and stomach in many respects is very marked, the two organs affording, as they do, the only channels through which the system receives its nourishment and communicates directly with the exterior. Both also being supplied as they are by the same nerve, must naturally perform their functions in a somewhat similar manner.

Your attention is called to the similarity of these organs to illustrate how natural it would be for the lungs to spasmodically contract, expel and withhold air, so long as the blood cannot absorb it. The pathology of the blood in the asthmatic is as yet very poorly understood and in fact very little has been written on the subject.

I am confident that there are chemical and pathological changes in the blood that cannot at present be scientifically described from lack of study and investigation of this subject, but it is to be hoped that this paper will provoke a discussion that will sufficiently interest all those who have an opportunity to study the blood in asthmatics, to cause them to make a thorough scientific work of it, and that each and all will decide to contribute something, so that in a short time our text-books will contain some positive and useful information in regard to this torturing affliction.

The most marked and observable pathological conditions of the blood in the production of asthma are the anemic, leukemic and lithemic types. Each of these types is capable of producing asthma, but quite frequently they are joint coadjutants. Then, again, in some cases first one type then another will be the predominant etiological factor. The etiology of asthma under these diverse abnormalities is extremely interesting and varied. Indeed, the etiological factors are so numerous and varied that it would be useless and next to impossible to enumerate all of them. Only those most evident and often proved will here be considered. While the different types differ greatly as to their etiology in many respects, yet they have numerous, common factors, but for various reasons it is here deemed best to describe the etiology with its variations under each type.

I found in looking over a history of 223 cases by Salter, that 129 were described as being thin, pale and emaciated; 44 very stout; 30 healthy looking; 20 not stated. I therefore conclude that out of his 223 cases, 129 or over half were of the anemic variety; 44 of the lithemic and 20 doubtful as to class.

My experience corroborates this record as a majority of my cases have been of the anemic variety, especially among adults. I will first consider the anemic type, through the history of a very interesting case. I would like you to observe in the history of this case the absolute freedom this patient enjoyed from all asthmatic symptoms, during such occupation or modes of life as tended to produce a healthy, robust and vigorous blood.

CASE I.—John H., occupation variable; aged 29; born New York State. Average height and weight, and usually of a healthy appearance. Inherited tendency from father's side, grandmother and one uncle having had asthma for a greater part of their lives. No history of tuberculosis. Majority of ancestors have attained an active, robust old age. Patient never sick from any other disease. First attack at age of 6, which lasted about a month. Had slight attacks at long intervals, but did not suffer much from asthma until aged 10, when the attacks became very frequent and quite severe, so that a physician was consulted, who prescribed Fowler's solution, which was pushed to its physiological limit, potassium and ammonium iodide, dissolved in grindilla robusta, with a small amount of ipecac and belladonna. This treatment, with slight variations, was followed for about three months (treatment I learned from the physician himself). After this treatment the asthma entirely disappeared and the patient did not even have a symptom, with two exceptions, which I will note, until the age of 22, twelve years after. The exceptions alluded to were, first, a mild attack of one night's duration at age 19, while attending an academy; attack occurred just before the commencement exercises of his graduating year, when he was reduced in health, pale and thin, from study, worry and sedentary life. Second mild attack of two nights' duration, aged 21, while teaching school.

During this twelve years of freedom from asthma, patient states that he was most decidedly of a sanguine temperament, being red-faced, of an erect posture, full-chested, very active, large and muscular for his age. Patient having been brought up on a farm, states that this period of his life was full of hardships, exposure and exertion, as he worked on the farm summers and attended or taught school winters. Four months of this time patient enjoyed the honor of being a house-to-house book canvasser, in a foreign state and among entire strangers, where it was his province to occupy a different bed nearly every night, the "spare" bed, a damp, poorly aired affair, in a cold room. Yet he always had robust health, undisturbed sleep and a voracious appetite. Diet always consisted largely of meats.

After this period of twelve years' rest, first genuine attack occurred at age of 22, after a twenty-four hours' trial at railroading. Patient took his first and last trip on a freight train from Hornellsville to Susquehanna and return, a continuous trip which took one day and a night. It was late in the fall; the day was warm, but the night was cold and frosty. Patient acted in the capacity of a full-fledged brakeman, as gang was one man short, riding most of the time, day and night, on top of the cars. Once during the night he came very near falling between the cars and being crushed to death. The fright added to the long continued nervous strain, loss of sleep and insufficient food, caused him to turn pale as a sheet. This anemic condition, as it proved to be, from fright and exhaustion, did not leave him, for when he reached home, some hours after, his mother knew that something had happened from his pale and haggard appearance. The following day after six hours peaceful slumber he went about his usual vocation, feeling as well as ever, not giving his fright a thought nor relating it to any one, and his nerves were as quiet and steady as ever, but all those he met remarked at his pale, haggard appearance. On the following night he was taken with a severe attack of asthma, which lasted about three days and nights. Patient describes this as one of his most severe attacks. A physician was called, not the one before consulted, who gave the usual remedies for asthma, excepting the arsenic, which did little or no good. Patient has had asthmatic attacks ever since, but as the history clinically proves a point which I wish to make, I shall rather minutely continue it. The following year patient taught school. During the full term the anemia continued, with loss of appetite, indigestion and a poor boarding house. Attacks were mostly nocturnal, following days of an unusual amount of work and worry. Would also come on if patient slept in a poorly aired bed, in a cool, damp room. There seemed to be no other exciting cause. Winter term found patient in a different boarding house. In fact, it was with a private family, who made cooking an art, and were rich livers. At every meal there were meats of various kinds, daintily prepared and perfectly cooked. Patient was soon restored to his usual, voracious appetite, which he most greedily satisfied. His red face reappeared, and although he slept in a cold room, underwent a great amount of exposure, and often caught cold, he did not have an attack nor a symptom of asthma that winter. Spring term found patient in another boarding house, furnished with a spacious, sanitary bed room, heat and ventilation being perfect, but the food was decidedly poor as to quality, quantity and mode of preparation. Patient's red face and appetite was soon replaced by indigestion, anemia and asthma. Attacks were nocturnal, coming on after days of unusual exhaustion. For several weeks there seemed to be no other exciting cause, but after this vicious circle became more firmly established, the exciting causes, or agents which usually produce paroxysms, were exceedingly numerous, in fact I never have read of a cause that was not mentioned in this case and many were described that I never before heard of. I did not, however, give them much consideration, for I believe that after a certain vicious circle has been established, the agents which excite asthmatic paroxysms are misleading, and that they only serve as an open trap into which most investigators have fallen.

The next three years of our patient's life we find to be occupied attending college in Buffalo, and the history repeats itself, hearty food, voracious appetite, no asthma; poor, insufficient food, anemia and asthma. For one whole year of college life, patient did not have an asthmatic symptom. Did not take much medicine while in college, but did have nose examined and exostosis was found and removed, but it never seemed to influence the asthma at all.

The last three years of patient's existence figures with office work and the upbuilding of a practice in his chosen profession. For first six months the patient lived in his office alone, taking two meals out and getting his own breakfast. The confinement, anxiety and insufficient food produced extreme anemia, with severe and frequent paroxysms of asthma, in fact patient states that he had the asthma so much that he could not think of any exciting cause, as it seemed to be one continuous attack. He was constantly under a physician's care and all asthmatic remedies were faithfully tried with no improvement. The only remedy that would allow sleep was chloroform.

At the close of this six months the patient's parents came to keep house for him, and he had such foods as a mother only can prepare. No one else could cook meat just to suit the taste, and always just the kind he wanted. As a result, patient lived almost exclusively upon a meat diet—meat three times a day—although this was just the opposite of what his physician had prescribed. But how much faith could he, and ought he to have had in physicians after this six months' trial? He not only went directly against his physician's orders, but stopped all medicines. As a result, his anemia and asthma disappeared, color came back to his face, and flesh to his bones. He continued to physically thrive for about one and a half years, with only a few mild attacks that were of no account. Patient was now so encouraged over his case that he thought he could be entirely cured. He therefore sought the advice of another physician, one of some repute in asthmatic cases. The physician, in obtaining his history, found that the patient was a heavy meat eater. From what he had read and knew of the subject the physician concluded that this was wrong, therefore meat was again interdicted, and patient put on a diet consisting mostly of milk, eggs, toast, and the like. He was also medicinally put on what I would consider an antiuric acid treatment, taking such medicines as syrup of hydriodic acid and sodium phosphate, alternating with lithium, potassium and sodium bicarbonate, also took a pill composed of arsenic, extract belladonna, extract physostigma, extract euphorbiæ piluliferæ fluid, extract sumbul and extract eucalyptus, also using a spray; in fact, from all that has been known on the subject, the treatment would be considered perfect. For about six months of this treatment the patient continued about the same; did not have much asthma when he began the treatment, and remained about the same for six months. At this time the patient married, also had more work than he could attend to, was so busy that he could not have his meals on time, and then often was compelled to hurry through with them. Three months of this life, in spite of the fact, or rather as I believe, hastened by the fact that the patient faithfully carried out his physician's directions, his vicious circle was again restored; improper food, indigestion, anemia, asthma. One feature that strongly suggests anemia in this case is that the attacks came on about eight or nine o'clock every evening. It seems that the blood would recuperate enough during the night's rest to last the patient for fifteen or sixteen hours of exertion, when the blood would again become so changed, that it could not properly absorb or carry oxygen, and as a result the patient had asthma. In a short time after retiring for the night the patient would drop into a sound, natural slumber, without the aid of any drug, and sleep until called in the morning, whether it were seven, eight or nine o'clock. On arising he would feel in good health and spirits until evening, when he would become so pale, haggard, exhausted and asthmatic that life was a burden.

The above history is of value in that it is a clinical proof of a case of asthma due to anemia or an abnormal blood which had undergone such chemical or physical changes that it could not absorb or carry a sufficient amount of oxygen; also that the nerves, reflexes and lungs took no part in the production of this asthma other than the performance of their physiological duty.

I was called to see the patient at about 8.30 o'clock in the evening, when I found him in paroxysms of asthma so severe that he could hardly speak even in monosyllables. I did not try to elicit any history that evening, but gave the patient a hypodermic injection of morphine, one-fourth, atropine, 1-300, nitro-glycerin, one one-hundredth and strychnine, one-sixtieth, and advised him to go to bed and sleep well, for I would call in the morning and wanted him to be able to furnish me with a complete history. Morning found the patient in good spirits after an undisturbed night's slumber, when I proceeded to elicit from him the above complete and interesting history.

After a thorough study of history I concluded that this case of asthma must be due to some blood dyscrasia. I, therefore, had a careful analysis of the blood made with the following results: Red blood corpuscles, 80 per cent.; hemoglobin, 40 per cent.; plasmodium malariæ, present.

After this analysis, the anemia was proven and a very important discovery made, that of the plasmodium malariæ. The treatment now was simple—kill the plasmodium and cure the anemia. I did not see the patient the following day until 4 o'clock p. m., and in order to prevent an attack of asthma that night, I immediately gave him twenty grains of quinine at one dose, and prescribed whiskey to be taken an hour later. That night the patient instead of sitting in his office as usual and laboring for air, had no signs of asthma and no bad effects from the quinine other than dryness of the throat. It seemed to check the secretions and stop the cough so suddenly as to give it the effect of a large dose of morphine and atropine. There being no cough nor dyspnea, it made the patient feel so light and free that his wife could not persuade him to remain at home that evening, so he walked half a mile to a political meeting, took part in the meeting and walked back home, feeling like a different man.

Patient called at my office next day, and I prescribed iron, arsenic, strychnine and quinine in large doses. I also gave him tincture of gentian compound with syrup of hypophosphites. As to diet, I ordered rare beef steak or roast, as much as he could eat and digest, and at every meal if he could tolerate it. The beef agreed with him to such an extent that he longed for it at every meal and would not eat without it. He took bone marrow also with excellent results. The patient was soon put on cold water baths, beginning with salt rubs. The rubs were given by soaking towels in a saturated solution of salt, allowing them to dry, then rubbing down with one every morning for ten mornings. After this, a cold plunge was taken every morning. The results of this treatment were phenomenal. The asthma did not reappear, but patient had a few symptoms for a week or so, and whenever a symptom presented itself the prophylactic measures, which never failed, consisted in large doses of quinine, followed by whiskey. After two months of this treatment, the patient regained his long lost sanguine temperament and ten pounds in weight.

This treatment works equally as well in asthma, due to anemia when the plasmodium malariæ is not found, and it is not probable that this germ figured for more than a few weeks in the history of the above case.

CASE II.—*Asthma Due to Leukemic Blood.*—Milton S., aged 35, born in Ohio, resides in Depew; weight, 153 lbs.; height, 5 feet 4 inches. Chest examination negative. No history of tuberculosis or asthma in ancestors. Occupation, carpenter. Ten years ago had syphilis. Since having syphilis, has been the father of four children, all of whom are now living and healthy. Never was sick until he had asthma, one year ago last July, when he consulted me. I did not know the nature of his asthma, and he not having money to pay for a blood analysis it was not then made. I then discovered that patient had been out of employment for nearly six months and that he and his family were in destitute circumstances, not having had a good wholesome meal in that time. I told patient that all I could do for him would be to give him a letter to the doctor for the poor and poor master, which he very reluctantly accepted. From July to December patient was under the care of this physician. In December the father-in-law of the patient called at my office and offered to go security for my bill, if I would take charge of the case, as the poor doctor had given up the patient. I therefore called and was informed that the patient had had asthma almost continually since I first saw him six months before, and during much of the time the only position in which he could breathe at all was standing with hands fastened to some object above his head. For the last month patient had had hemoptosis, coughing up so much blood at one time that his wife thought he would bleed to death. The patient's family and Poor Physician thought he had tuberculosis and that he would live for only a short time. Examination of heart, lungs and kidneys negative, or at least, as I thought, only such conditions existed as were to be expected from such a protracted and severe attack of asthma. Poor Physician informed me that he had faithfully tried all asthmatic remedies, and especially potassium iodide, as he expected the syphilis had something to do with the case, but with no benefit. I became interested in the case and ordered patient to be taken to a hospital immediately. The family objected, as they had not the financial means, neither did they deem it any use. However, by persistence I learned that the patient had a well-to-do brother in Ohio. I obtained the brother's name and address and wrote to him explaining the situation, also giving my opinion of the case. As a result the patient was sent to a hospital in Cleveland, Ohio. I accompanied him to the hospital and had his sputum and blood analysed, with the following results: Leucocytes, 1 to 19; red corpuscles, about normal as to number; hemoglobin, greatly diminished; plasmodium malariæ, absent; sputum analysis, B. T. absent; Curshmann's spirals, absent; Leyden's crystals, present.

After blood analysis, the patient was given a careful general examination. The spleen was decidedly enlarged. Nothing unusual was found about the heart and kidneys for a patient who had had a six months' attack of asthma. Lung examination revealed all the characteristic symptoms of asthma. After the above examinations we concluded that we had a case of asthma, due to leukemic blood, and that the most probable cause of the patient's leukemic condition was his poor food, unsanitary surroundings and syphilis.

The treatment consisted in first depleting the system as far as strength of patient would allow, the object being to drain the lymphatic system and carry off the excessive amount of lymph. This was accomplished by calomel, followed by Epsom salts. During this draining process, which lasted twenty-four hours, the patient was sustained by a large amount of whiskey, liquid peptonoids, strychnine and predigested milk. The quantity of lymph that was thus extracted was surprising. The patient became so weak that it was necessary to apply artificial heat, and to give copious injections of saline solution. These injections were given subcutaneously in region of the nipples, by means of the needle and hose of an aspirating apparatus attached to a fountain syringe. After forty-eight hours of this treatment patient began to rally, sat up in bed and remarked how well he felt and easily he breathed. In fact his asthma had nearly disappeared, and it did not again manifest itself to any appreciable extent. The rest of treatment consisted in large doses of Fowler's solution, strychnine and a rich nutritious diet.

After three months of this treatment the patient returned home, obtained employment at his trade and has not been sick since, and with the exception of a few mild attacks, the only reminder of his asthma is shortness of breath after unusual exertion.

CASE III.—*Asthma Due to Lithemic Blood.*—Mrs. R., aged 38; height, 5 feet 4 inches; weight, 180 lbs.; born in New York; resides in Depew; married at age 18; mother of ten children, all living and healthy. One cousin and two brothers have asthma. No history of tuberculosis. Patient had rheumatism two years ago, which confined her in bed three weeks, otherwise never sick with anything but asthma; has had asthma for thirteen years. The patient stated that she had not been able to sleep scarcely one whole night in bed during this thirteen years. She also stated that her asthma continued to grow worse, and that for three or four weeks before treatment had one continual attack. She was always a hearty meat eater. Claims that she had been treated for years with no benefit. The principal exciting causes were east wind, damp, stormy weather, wet feet and exertion.

I was called to see the patient one year and a half ago—a night call. I found her sitting up, gasping for breath and unable to speak. Her husband was greatly excited, expecting her to die every minute, and wanted to send for the priest. After an examination of her pulse, heart and lungs, I told him he need not be alarmed, and immediately gave her a hypodermic injection of strychnine one-thirtieth, morphine one-fourth, atropine one-three-hundredth and nitro-glycerin one-hundredth, also gave her by mouth twenty grains of quinine and a tablespoonful of Epsom salts, dissolved in half a glass of hot water. I watched the patient until she began to breathe easier, then advised her to lie down, as I believed she would be able to sleep. The next morning I found her in good condition, and learned that she had slept fairly well the previous night. The only thing the patient complained of was ringing in the ears and dryness of throat. She was also alarmed at the fact that she could not raise anything when she coughed, but after informing her that I intended to draw off through her kidneys and bowels the irritating substances that had produced the mucus she had coughed up she was quite at rest. Analysed the urine with the following results: Specific gravity, 10.24; reaction, very acid; albumin, absent; sugar, absent; uric acid, increased.

*Blood Analysis.*—Red corpuscles, normal; white corpuscles, normal; alkalinity, diminished; plasmodium malariæ, absent.

This case proved to be a very discouraging one to manage. For a long time treatment seemed to be of but little or no benefit. Approaching storms, wet feet, exertion, overheating, or a sudden chilling, seemed to reduce the alkalinity of the blood, thus allowing a fresh precipitation of uric acid and other crystals, which continued to act as an irritant, sufficient to interfere with the capillary circulation of the lungs. This impaired lung capillary circulation soon resulted in poorly oxygenated blood, which in turn irritated the respiratory center of the medulla oblongata, as poorly oxygenated blood always must, or respirations would cease. Thus the lungs were encouraged or compelled to put forth vigorous respiratory efforts, but the blood not being there to absorb or carry the oxygen, the lungs soon became exhausted from their useless effort, and poorly nourished condition, and as a result they, as the most natural and only thing to do, spasmodically contracted.

After about six weeks' treatment the asthma began to yield and one month later the patient thought she was cured. This was about one year and two months ago, and since that time the patient has had only a few mild attacks. The attacks were so mild that she has been able to sleep in her bed every night and all night since treatment.

In this case most every therapeutic measure was tried, but those which, as I believe, produced the cure, consisted of a milk and buttermilk diet, with eggs, toast, bread, preserves, and the like; also sodium phosphate, potassium iodide, latter often given in form of syrup hydriodic acid; alternating with potassium, sodium and lithium bicarbonate. The following pill was also given throughout the treatment with a few changes every twenty days:

| | | |
|---|---|---|
| ℞ | Strychniæ sulphate | |
| | Acid arsenosi | āā gr. ii. |
| | Ext. euphorbiæ piluliferæ fl | ℥ iii. |
| | Aurii et sodii chloridi | |
| | Zinci phosphatis | āā gr. iij. |
| | Ext. belladonnæ | |
| | Ext. physostigmati | āā gr. v. |
| | Aloes socotrinæ | gr xii. |
| | Quinine sulphatis | ℥ iii. |
| | Ext. sumbul | |
| | Ext. eucalyptis | āā ℥ ii. |

M. et ft. pil. no lx. Sig. One before food.

This made a rather large pill, but each ingredient was prescribed with an object in view and the patient was easily encouraged to take it, which she did with splendid results. Thus the alkaline treatment eliminated the uric acid and other irritating crystaline substances from the blood, while the pill restored the lungs, nerves, heart and alimentary tract back to their normal standard.

---

AT THE Maternité of Marseilles, the mortality amongst infants born before term has diminished considerably since 1896. In that year there were thirty-six deaths, while in 1897 there were six deaths only. This notable decrease seems to be due to judicious employment of artificial serum in enemas at the daily dose of three ounces, or an ounce three times a day. Subcutaneous injections are rarely employed.—*Medical Age.*

